# Association between cannabinoid 1 receptor availability and glutamate levels in healthy controls and drug-free patients with first episode psychosis: a multi-modal PET and 1H-MRS study

**DOI:** 10.1007/s00406-020-01191-2

**Published:** 2020-09-28

**Authors:** Faith Borgan, Mattia Veronese, Tiago Reis Marques, David J. Lythgoe, Oliver Howes

**Affiliations:** 1 Psychosis Studies Department, Institute of Psychiatry, Psychology and Neuroscience, King’s College London, London, England; 2 Institute of Clinical Sciences, Faculty of Medicine, Imperial College London, Hammersmith Hospital, London, W12 0NN UK; 3 Centre for Neuroimaging Sciences, Institute of Psychiatry, Psychology and Neuroscience, King’s College London, London, England

**Keywords:** Glutamate, Cannabinoid, Endocannabinoid system, Cannabinoid 1 receptor, Schizophrenia

## Abstract

Cannabinoid 1 receptor and glutamatergic dysfunction have both been implicated in the pathophysiology of schizophrenia. However, it remains unclear if cannabinoid 1 receptor alterations shown in drug-naïve/free patients with first episode psychosis may be linked to glutamatergic alterations in the illness. We aimed to investigate glutamate levels and cannabinoid 1 receptor levels in the same region in patients with first episode psychosis. Forty volunteers (20 healthy volunteers, 20 drug-naïve/free patients with first episode psychosis diagnosed with schizophrenia/schizoaffective disorder) were included in the study. Glutamate levels were measured using proton magnetic resonance spectroscopy. CB1R availability was indexed using the distribution volume (V_T_ (ml/cm^3^)) of [^11^C]MePPEP using arterial blood sampling. There were no significant associations between ACC CB1R levels and ACC glutamate levels in controls (*R* = − 0.24, *p* = 0.32) or patients (*R* = − 0.10, *p* = 0.25). However, ACC glutamate levels were negatively associated with CB1R availability in the striatum (*R* = − 0.50, *p* = 0.02) and hippocampus (*R* = − 0.50, *p* = 0.042) in controls, but these associations were not observed in patients (*p* > 0.05). Our findings extend our previous work in an overlapping sample to show, for the first time as far as we’re aware, that cannabinoid 1 receptor alterations in the anterior cingulate cortex are shown in the absence of glutamatergic dysfunction in the same region, and indicate potential interactions between glutamatergic signalling in the anterior cingulate cortex and the endocannabinoid system in the striatum and hippocampus.

**Electronic supplementary material:**

The online version of this article (10.1007/s00406-020-01191-2) contains supplementary material, which is available to authorized users.

## Introduction

Schizophrenia is ranked as one of the most disabling health conditions [1], with annual costs ranging between 94 million to 102 billion dollars across a range of countries [2]. While current pharmacological treatments predominately block the D2 dopamine receptor [3], they have limited efficacy in reducing symptom severity in 30% of patients [4, 5] and they fail to improve cognitive deficits [6, 7]. In view of these limitations, further work is needed to identify therapeutic mechanisms and biomarkers that may be useful for targeting both psychotic and cognitive symptoms in schizophrenia [8–10].

The pathophysiology of schizophrenia has been postulated to involve glutamatergic dysfunction [11, 12], since N-methyl-D-aspartate receptor (NMDAR) antagonists induce positive and negative symptoms and cognitive impairments in healthy controls, mimicking the phenotype of schizophrenia [13–15]. In particular, low doses of ketamine, an NMDAR antagonist, induce psychotic symptoms, cognitive impairments, and increase anterior cingulate cortex (ACC) glutamate levels in healthy volunteers [16]. While this finding was taken to suggest that altered ACC glutamate levels may be associated with the induction of psychotic symptoms, other studies have shown that ketamine induces psychotic symptoms in the absence of any change in ACC glutamate levels [17, 18].

Glutamate levels can be measured in vivo using proton magnetic resonance spectroscopy (1H-MRS). Discrepant findings have been reported in chronic patients taking antipsychotic medication including reports of no differences [19] and decreased glutamate levels in the anterior cingulate cortex (ACC) [20, 21]. In contrast, patients with first episode psychosis who are not taking antipsychotics have largely been shown to have no differences in ACC glutamate levels relative to healthy volunteers [22–25], with the exception of one study reporting decreased ACC glutamate levels [26]. Despite the absence of group differences in ACC glutamate levels in drug-free patients with first episode psychosis, ACC glutamate levels have been shown to be negatively associated with symptom severity and striatal dopamine synthesis capacity [27].

Excitatory and inhibitory synaptic transmission is regulated by the cannabinoid 1 receptor (CB_1_R), a G-protein-coupled receptor distributed on pre-synaptic nerve terminals of glutamate and GABA interneurons throughout the cortex, hippocampus, and striatum [28]. CB_1_R activation transiently suppresses neurotransmitter release by the inhibition of N-, P-, and Q-type calcium channel openings and through activation of inwardly rectifying potassium channels on the pre-synaptic neuron [29–31] and adjacent neurons containing CB_1_R within a within < 20 μm radius [29, 32]. However, these effects can be blocked by CB_1_R antagonists or the genetic ablation of the CB_1_R [33].

Since CB_1_R activation inhibits synaptic transmission at both glutamate and GABA neurons in the ACC, CB_1_R activation regulates both excitatory and inhibitory synaptic transmission. In particular, CB_1_R activation at GABAergic interneurons in the ACC disinhibits downstream synaptic transmission [34], whereas CB_1_R activation on glutamate neurons in the ACC inhibits glutamate release [35]. These findings indicate that ACC CB_1_R regulate both excitatory and inhibitory synaptic transmission.

Previous literature has shown that CB_1_R agonists modulate glutamate release in rodents [36, 37] and humans [38]. Moreover, mice with CB_1_R deletions on cortical glutamate neurons exhibit enhanced excitability on cortical glutamate neurons leading to stronger seizures following the kainic acid induced excitation in hippocampal pyramidal neurons as well as the significant loss of glutamatergic neurons [39, 40].

Since we recently showed that drug-free patients with schizophrenia have lower levels of CB_1_R in the ACC, and that greater reductions were linked to greater cognitive deficits [41], we specifically aimed to investigate whether CB_1_R alterations in the ACC may be associated with glutamatergic alterations in the ACC in the same drug-free patients. We hypothesized that ACC CB_1_R levels would be associated with ACC glutamate levels. We also aimed to conduct exploratory analyses to investigate the association between ACC glutamate levels and CB1 receptors in other brain regions where CB_1_R levels are altered in schizophrenia [41–43]. We also aimed to investigate the association between ACC glutamate levels, symptom severity, and cognitive functioning, as determined by the Wechsler Digit Symbol Coding test [44] based on evidence that it is highly corelated with total cognitive deficits in first episode psychosis [45].

## Methods

### Ethics statement

Relevant ethical approvals were obtained from the Camberwell St. Giles Research Ethics Committee (14/LO/1289). Informed written consent was obtained from all subjects prior to participation.

### Participants

A total of 40 volunteers (20 healthy volunteers and 20 drug-free patients with first episode psychosis) were recruited. All patients were recruited from early intervention services for psychosis in London, United Kingdom. Age (age ± 3 years) and sex-matched healthy volunteers were recruited via local advertising.

### Inclusion/exclusion criteria

First episode psychosis patients met the following inclusion criteria: (1) diagnosis of a psychotic disorder as determined by the ICD-10; (2) antipsychotic naïve or antipsychotic free for at least 6 months, as determined by medical records and self-report; (3) no current use of any concurrent pharmacological treatments as determined by medical records and self-report (e.g., benzodiazepines, antidepressants etc.); (4) illness duration of less than 3 years as determined by medical records and self-report.

Healthy volunteers met the additional inclusion criteria including: (1) no current or lifetime history of an Axis-I disorder as determined by the Structured Clinical Interview for DSM-IV-TR (SCID-I/P) [46] and (2) no family history of an Axis-I disorder in first- and second-degree relatives as determined by the Family Inventory for Genetics studies (FIGS) [47]. Exclusion criteria for all volunteers: (1) reported current use of any recreational substances within the last month; (2) screened positive on a urine toxicology test that can detect THC metabolite THCCOOH for up to 30 days (50 ng/ml cut off) (SureScreen, Diagnostics; 50 ng/ml cut off); (3) screened positive on a multi-panel drug screen detecting the following substances: amphetamine (300 ng/ml cut off), cocaine (150 ng/ml cut off), ketamine (1000 ng/ml cut off), methamphetamine (300 ng/ml cut off), and opiates (2000 ng/ml cut off) (SureScreen Diagnostics).

Volunteers were aged 18–35 and were able to give informed, written consent. Volunteers were excluded if they had: (1) a history of a head injury leading to loss of consciousness, (2) personal or family history of neurological or physical health problems, (3) contraindications to MRI safety including head trauma, pregnancy, the presence of metal plates, pins, bridges, or dentures; or 4) current history of substance use or dependence as determined by the Structured Clinical Interview for DSM-IV-TR (SCID-I/P) [46].

### Demographics

Age, sex, ethnicity, and years of education were recorded.

### Clinical measures

Clinical symptom severity was determined by trained staff using the Positive and Negative Syndrome scale (PANSS) [48]. Social and occupational functioning was measured using the Global Assessment of Functioning (GAF) [49]. Healthy volunteers were screened for personal and family history of mental health problems using SCID-I/P [46] and FIGS [47]. Cognition was measured using the Wechsler Digit Symbol Coding test [44] based on evidence that it is highly corelated with total cognitive deficits in first episode psychosis [45].

### Neuroimaging

#### Positron emission tomography

CB1R-selective radiotracer, [^11^C]MePPEP, was used to investigate the distribution volume of the cannabinoid 1 receptor. A continuous 90-min PET scan acquired using a Hi-Rez Biograph 6 CT44931 scanner in three-dimensional mode, following a bolus injection (mean = 314; SD = 34.4 MBq) of [^11^C]MePPEP synthesized using methods reported elsewhere [50, 51]. CT scans were acquired prior to each PET scan for correction for attenuation and scatter. Continuous arterial blood sampling took place for the first 15 min of the scan which was followed by discrete blood sampling at 2, 5, 10, 15, 20, 25, 35, 40, 50, 60, 70, 80, and 90 min after the radioligand injection. Images were reconstructed with filtered back projection including corrections for attenuation and scatter. PET scans were performed between 9:00 AM and 3 PM; participants fasted (water was allowed) and abstained from alcohol and substances for more than 12 h before undergoing the scan. PET and MRI scans were conducted approximately 2 weeks apart.

#### Magnetic resonance imaging and proton magnetic resonance spectroscopy (1H-MRS)

**Fig. 1:**
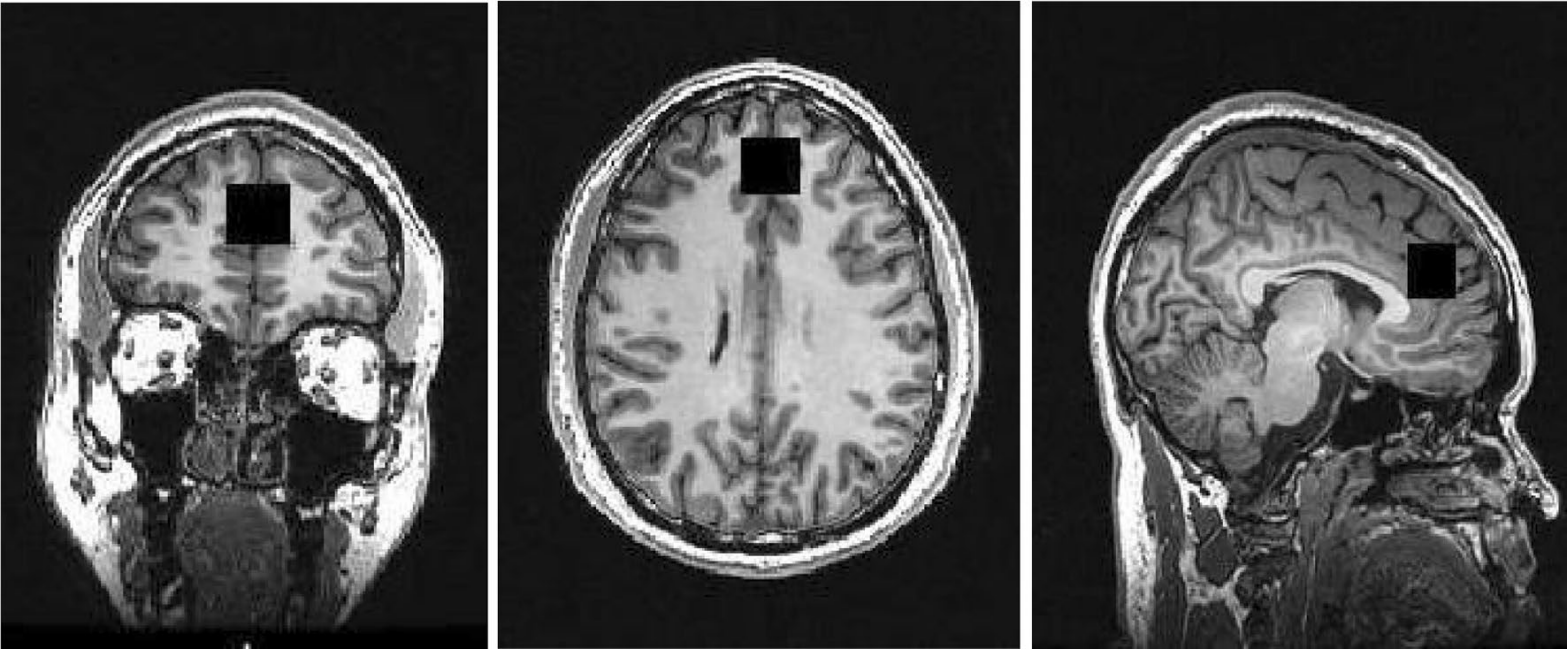
Coronal (left), axial (middle), and sagittal (right) planes depicting the placement of the voxel over the anterior cingulate during the proton magnetic resonance spectroscopy scan. The placement of the anterior cingulate voxel was based on the midline sagittal localizer with the centre of the 20 mm × 20 mm × 20 mm voxel placed 13 mm above the anterior portion of the genu of the corpus callosum, perpendicular to the anterior commissure–posterior commissure line to minimize the inclusion of white matter and cerebral spinal fluid

MRI scans were acquired using the General Electric MR750 3.0 T MRI scanner. High-resolution 3D T1-weighted structural MRI images were acquired for the 1H-MRS voxel prescription and anatomical co-localisation (in-plane matrix size of 256 × 256, FOV = 26.0 mm) using a whole-brain, sagittal IR-SPGR acquisition, and an eight-channel head coil (TR = 7.34 mm, TI = 400 ms, inversion time = 4 s, flip angle = 11°, and slice thickness = 1.2 mm). Data included here were previously published as a part of a multi-centre study [41]. The neuroimaging acquisition parameters for the acquisition of the proton magnetic resonance spectroscopy data were as follows: internal localizer scans were used to determine the anterior commissure–posterior commissure line and inter-hemispheric angle. For the voxel placement, the sagittal IR-SPGR scans were reformatted into axial and coronal planes and the voxel positioned in the ACC. This was followed by auto pre-scans for optimisation of water suppression and shimming of the MRS voxel. 1H-MRS spectra were acquired from the anterior cingulate region-of-interest (right-left 20 mm × anterior–posterior 20 mm × superior–inferior 20 mm). The placement of the anterior cingulate voxel was based on the midline sagittal localizer with the centre of the 20 mm × 20 mm × 20 mm voxel placed 13 mm above the anterior portion of the genu of the corpus callosum, perpendicular to the anterior commissure-posterior commissure line to minimize the inclusion of white matter and cerebral spinal fluid (CSF) (see Fig. 1 for images of the coronal, axial, and sagittal placement of the voxel). Finally, the ^1^H-MRS spectra (Point RESolves Spectroscopy (PRESS), TE = 30 ms, TR = 2 s) were obtained through the PROton Brain Examination (PROBE) sequence by GE, which includes water suppression. Water unsuppressed scans were also acquired for subsequent eddy current correction and water referenced metabolite quantification. A subset of these data (17 subjects) were reported previously [25]. As described previously [52], MRI scans were performed between 9:00 and 11:30 am; participants fasted (water was allowed) and abstained from alcohol and other substances for more than 12 h before undergoing the MRI scan.

## Analysis

### Imaging analysis

#### Positron emission tomography

Methods were reported previously [41]. Data pre-processing was performed using a combination of Statistical Parametric Mapping 12 (https://www.fil.ion.ucl.ac.uk/spm) and FSL (https://www.fsl.fmrib.ox.ac.uk/fsl) functions, as implemented in MIAKAT Version 1.0 (miakat.org) using MATLAB R2015b [53]. Motion correction was applied to non-attenuation corrected images [54]. Non-attenuated-corrected frames were realigned to a single “reference” frame by employing a mutual information algorithm. The transformation parameters were then applied to the corresponding attenuated-corrected dynamic images, creating a movement-corrected dynamic image which was used for the analysis. Realigned frames were then summated to create single-subject motion-corrected maps which were then used for MRI and PET co-registration prior to PET data quantification. T1-weighted structural images were co-registered to the PET image using rigid body transformation. Normalization parameters were obtained by warping the co-registered structural MRI to MNI space (International Consortium for Brain Mapping ICBM/MNI) using bias-corrected segmentation. The inverse of these parameters was used to fit a neuroanatomical atlas to each individual PET scan using the Hammersmith atlas [55]. Whole blood time–activity curves (TACs) were fitted using a multi-exponential function as derived by Feng’s model [56]. For each scan, a time delay was fitted and applied to the input functions (both parent and whole blood TACs) to account for any temporal delay between blood sample measurement and target the tissue data. Voxel-based morphometry analyses reported elsewhere [41] indicated that there were no significant volumetric differences between patients and controls in the anterior cingulate cortex, and thus, no partial volume corrections were conducted.

CB_1_R distribution volume (V_T_) was calculated using the Logan graphical method with a metabolite-free arterial plasma input function [57] using a regional approach. An independently derived region of interest of the anterior cingulate cortex was obtained from a standard probabilistic atlas [55]. A 3D image containing the MNI coordinates used for the MRS voxel placement was also generated using SPM12, so that ACC V_T_ values could be extracted from the ACC MRS voxel using MATLAB R2015b [53]. The ACC was selected given prior findings implicating this region in the pathophysiology of psychotic and cognitive symptoms [58, 59], and findings indicated that the CB_1_R regulates synaptic transmission in this region [29, 60].

#### Proton magnetic resonance spectroscopy

Methods were reported previously [25]. Water-scaled metabolites were analysed using LC-model 6.3-0I using an experimentally acquired basis set to estimate concentrations of 16 metabolites including glutamate, glutamine, GLX (glutamate + glutamine), N-Acetyl-Aspartate (NAA), l-alanine, aspartate, creatine, phosphocreatine, GABA, glucose, glycerophosphocholine, glycine, myo-inositol, l-lactate, N-acetylaspartylglutamate, phosphocholine, and taurine. Metabolite analyses were restricted to spectra with linewidth (full-width at half-maximum; FWHM) ≤ 0.1 ppm, Cramér–Rao lower bounds (CRLB) for glutamate ≤ 20%, and signal-to-noise ratio ≥ 5. Corrections were applied to take into account the relative distribution of cerebrospinal fluid within the anterior cingulate. In-house scripts written in Python were used to identify the relative distribution of white matter, grey matter, and cerebrospinal fluid in the 8 cm^3^ voxel prescribed to the anterior cingulate. The following correction described previously [61] was subsequently applied to correct for CSF, grey matter (GM), and white matter (WM) content within the 8 cm^3^ voxel, where M = raw metabolite value:

$${\text{Mcorr}} = M \, (\frac{{{\text{WM}} + \left( {{\text{GM}} \times 1.22} \right) + \left( {{\text{CSF}} \times 1.55} \right)}}{{\left( {{\text{WM}} + {\text{GM}}} \right)}}).$$

Apart from assuming the T_2_ = 80 ms for tissue water, no corrections were applied for tissue and metabolite T_1_ and T_2_ relaxation. The equation also includes a correction for the default assumption the voxel is pure WM during the LCModel analysis Table 1.

**Table 1:** Demographics

	Healthy volunteers	First episode psychosis patients
*N*	20	20
Age (years) mean (SD)	Mean = 27.15; SD = 6.12	Mean = 27.00 SD = 5.06
Sex (male/female)	20/0	20/0
Ethnicity (CAUCASIAN/black African or Caribbean/Asian/mixed)	7/2/9/2	10/4/5/1
Diagnosis (schizophrenia/schizoaffective disorder)	N/A	18/2
Illness duration (months) mean (SD)	N/A	Mean = 22.66; SD = 11.64
Duration of prior treatment (if applicable) (months) mean (SD)	N/A	Mean = 4.21 SD = 5.44
Current use of antipsychotics (yes/no)	N/A	0/20
Prior use of antidepressant (yes/no)	N/A	5/15
Prior use of antipsychotics (yes/no)	N/A	16/4
PANSS positive mean (SD)	N/A	Mean = 26.95 SD = 17.75
PANSS negative mean (SD)	N/A	Mean = 22.79 SD = 6.96
PANSS general mean (SD)	N/A	Mean = 39.74 SD = 10.77
PANSS total mean (SD)	N/A	Mean = 84.21 SD = 22.10
PANSS five-factor positive mean (SD)	N/A	mean = 14.61 SD = 4.34
PANSS five-factor negative mean (SD)	N/A	Mean = 24.94 SD = 7.28
PANSS factor cognitive disorganization mean (SD)	N/A	Mean = 18.61 SD = 6.17
PANSS five-factor depression/anxiety mean (SD)	N/A	Mean = 8.50 SD = 2.94
PANSS five-factor excitability/hostility mean (SD)	N/A	Mean = 6.28 SD = 3.51
Current cannabis use (yes/no)	0/20	0/20
Current alcohol use (yes/no/missing data)	12/8/0	12/8/2

*N* sample size, *FEP* first episode psychosis, *t* independent samples *t* test, *χ*^2^ Chi-square, *df* degrees of freedom, *N/A* not applicable, *SD* standard deviation, *BPRS* brief psychiatric rating scale

All demographics reported in this table were reported previously [41]

### Statistical analysis

The Statistical Package for the Social Sciences (version 22) [62] was used for all statistical analyses. Data normality was assessed using the Shapiro–Wilk test.

Independent samples *t* tests were also used to investigate group differences in experimental PET variables that may potentially confound the results including weight, dose (Mbq), injected mass (µg), specific activity (GBq/μmol), fraction of ligand free in plasma (fp), and total motion (frame-to-frame displacement) (see Table 2) and experimental 1H-MRS variables that may potentially confound the results including linewidth, signal-to-noise, and data shift (see Table 3).

**Table 2:** Experimental PET variables

	Healthy volunteers	FEP patients	T	df	*p*
*N*	20	20			
Weight (kg)	*M* = 78.29; SD = 13.26	*M* = 85.09; SD = 14.17	− 1.568	38	0.13
Body mass index^a^	*M* = 25.47; SD = 3.78	*M* = 26.65; SD = 5.24	− 0.674	26	0.51
Dose (MBq)	*M* = 311.32; SD = 44.87	*M* = 311.50; SD = 27.48	0.016	38	0.98
Injected mass (µg)	*M* = 4.31; SD = 1.60	*M* = 4.72. SD = 2.46	− 0.583	38	0.56
Specific activity GBq/μmol	*M* = 97.32; SD = 287.91	*M* = 158.38; SD = 556.02	− 0.436	38	0.67
Fp (% if > 1 or fraction if < 1)	*M* = 0.19; SD = 4.68	*M* = 0.16; SD = 0.05	1.758	38	0.09
Total scanner motion (mm)^b^	*M* = 12.58; SD = 4.68	*M* = 13.78; SD = 5.95	− 0.709	38	0.48

*FEP* first episode psychosis, *N* number, *kg* kilograms, *mm* millimetres, *MBq* megabecquerel, *µg* microgram, *umol* micromoles, *GBq* gigabecquerel

^a^Body mass index, calculated using methods described previously [84]

^b^Total scanner motion was defined as the sum of total frame-to-frame movement during imaging acquisition

**Table 3:** Experimental 1H-MRS variables

	Healthy volunteers	FEP patients	*t*	df	*p*
*N*	20	20			
Linewidth (ppm)	*M *= 0.03; SD = 0.01	*M* = 0.04; SD = 0.01	− 1.257	38	0.22
Signal-to-noise	*M* = 26.35; SD = 6.47	*M* = 24.75; SD = 6.31	0.79	38	0.43
Data shift (ppm)	*M* = 0.02; SD = 0.01	*M* = 0.02; SD = 0.01	0.05	38	0.96

*ppm* parts per million

To test the main hypothesis that ACC CB_1_R availability would be associated with ACC glutamate levels, a Pearson’s correlation coefficient was calculated to investigate the association between ACC glutamate levels and ACC CB_1_R availability (using a standard probabilistic atlas [55] and values extracted from the whole ACC MRS voxel). Exploratory analyses were also conducted to determine whether ACC glutamate levels were associated with CB_1_R availability in the striatum, hippocampus, and thalamus.

Exploratory analyses were also conducted to investigate the association between ACC glutamate levels and cognition and symptom severity (PANSS total and PANSS 5-factor model described previously [63].

## Results

### Demographics

There were no significant differences between healthy volunteers and patients in age, gender, ethnicity, alcohol use, tobacco use, or history of cannabis abstinence in study 1 (see Table 1).

### Positron emission tomography: cannabinoid receptor availability

The CB_1_R imaging data from this cohort have previously been reported. As we previously reported [41], patients showed significantly lower CB_1_R availability in the anterior cingulate cortex relative to controls (Hedge’s g = 0.8).

### Proton magnetic resonance spectroscopy (1H-MRS)

The 1H-MRS data from this cohort have previously been reported. As we previously reported [25], there were no significant differences between healthy volunteers and patients with first episode psychosis in glutamate (see Table 4).

**Table 4:** 1H-MRS results

	Healthy volunteers	First episode psychosis patients	*t*	df	*p*
Glutamate	15.38 (1.85)	16.36 (1.90)	− 1.659	38	0.11

### Association between cannabinoid 1 receptor availability and metabolite levels

**Fig. 2:**
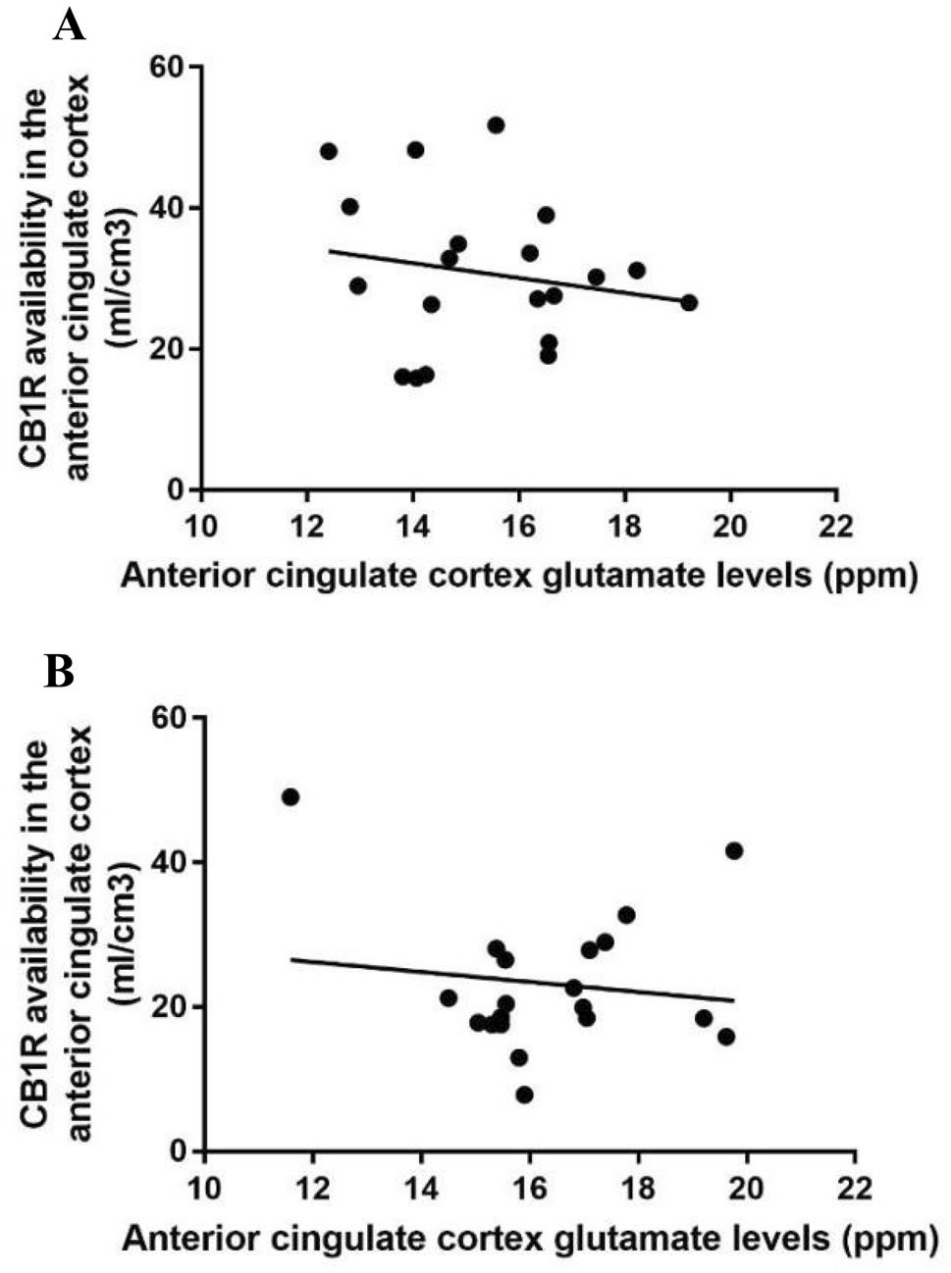
Scatter plot showing no association was detected between anterior cingulate cortex glutamate levels (ppm) and anterior cingulate cortex cannabinoid 1 receptor availability, indexed using the V_T_ of [^11^C]MePPEP in **a** healthy volunteers controls (*R* = − 0.24, *p* = 0.32) or **b** patients (*R* = − 0.10, *p* = 0.25)

There was no significant association between ACC CB_1_R levels and ACC glutamate levels in controls (*R* = − 0.24, *p* = 0.32) or patients (*R* = − 0.10, *p* = 0.97) (see Fig. 2). Since cannabinoid 1 receptor levels in the anterior cingulate cortex were extracted using a probabilistic atlas which may be different to the MRS voxel prescribed to the anterior cingulate cortex, we repeated these analyses using identical regions of interest to extract the PET and MRS data using the MRS voxel prescribed to the anterior cingulate cortex. Findings remained unchanged when investigating the association between ACC glutamate and V_T_ values extracted from the ACC MRS voxel. In particular, there were no significant associations between ACC glutamate and ACC V_T_ values in controls (*R* = − 0.055, *p* = 0.819) or patients (*R* = 0.086, *p* = 0.719).

Exploratory analyses in healthy volunteers indicated that ACC glutamate levels were negatively associated with CB_1_R availability in the striatum (*R* = − 0.50, *p* = 0.02), hippocampus (*R* = − 0.50, *p* = 0.042), but not the thalamus (*R* = − 0.30, *p* = 0.30) or the ACC (*R* = − 0.26, *p* = 0.32). In contrast, patients failed to show any associations between ACC glutamate levels and CB_1_R availability in the striatum (*R* = − 0.30, *p* = 0.20), hippocampus (*R* = 0.14, *p* = 0.60), thalamus (*R* = − 0.12, *p* = 0.61), or ACC (*R* = − 0.01, *p* = 0.98).

### Association between metabolite levels and symptom severity

There were no significant associations between total PANSS symptom severity and glutamate levels (*R* = − 0.10, *p* = 0.69), GLX levels (*R* = 0.05, *p* = 0.85), glutamine levels (*R* = 0.15, *p* = 0.55), or NAA levels (*R* = − 0.03, *p* = 0.90) in the ACC. These findings remained unchanged when investigating the association between PANSS subscales of positive, negative, and general symptom severity. Using a five-factor model [63], patients showed a significant positive association between glutamate levels in the anterior cingulate and the positive factor (*R* = 0.613, *p* = 0.007) (see supplementary Figure 1), the depression/anxiety factor (*R* = 0.514, *p* = 0.029) (see supplementary Figure 2) and a trend-level association with the cognitive/disorganization factor (*R* = 0.411, *p* = 0.09).

### Association between metabolite levels and cognition

There was no significant association between WAIS digit symbol coding test scores and glutamate levels in controls (*R* = − 0.18, *p* = 0.48) or patients (*R* = − 0.23, *p* = 0.34).

## Discussion

Our findings extend our previous work in an overlapping sample showing that cannabinoid 1 receptor levels are lower in anterior cingulate cortex in drug-naïve/free patients with first episode psychosis [41] to show that these cannabinoid 1 receptor alterations are shown in the absence of evidence of glutamatergic alterations in the same brain region. In contrast to our predictions, ACC glutamate was not associated with ACC CB_1_R availability in patients or controls. Exploratory analyses indicated that ACC glutamate levels were associated with CB_1_R availability in the hippocampus and striatum in controls, but not patients. We also showed that ACC glutamate levels were associated with greater symptom severity on the positive factor (*R* = 0.613, *p* = 0.007) and the depression and anxiety factor (*R* = 0.514, *p* = 0.029) using a five-factor model [63].This is the first study, as far as we’re aware, to investigate the association between cannabinoid 1 receptor levels and glutamate levels in humans or patients with schizophrenia.

These findings extend previous literature, showing that glutamate release can be modulated following the administration of a cannabinoid 1 receptor agonist in rodents [36, 37] and humans [38] to show that CB_1_R levels are related to glutamate levels in vivo in healthy individuals. These findings also extend previous work, showing that mice with CB1R deletions on cortical glutamate neurons exhibit enhanced excitability on hippocampal glutamate neurons leading to stronger seizures and neuronal loss [39, 40]. Given previous work indicating that CB_1_R are localised on glutamate neurons in the ACC [35], hippocampus [64], and cortical layer 5 [65], and that CB_1_R activation suppresses glutamate release via depolarisation-induced suppression of excitation (DSE), we expected an inverse relationship between CB_1_R and glutamate levels. Our finding that controls showed an association between ACC glutamate and CB_1_R availability in the striatum and hippocampus but not the ACC or thalamus partially support this, and highlight the need for future studies to understand endocannabinoid circuits. Our finding that patients failed to show any of the relationships between CB1R and glutamate seen in controls highlights how endocannabinoid signalling pathways involving the anterior cingulate, hippocampus, and striatum may be disrupted in schizophrenia.

Previous literature has shown that the endogenous CB_1_R agonist, AEA, that is elevated in schizophrenia [66] modulates synaptic plasticity by inhibiting glutamatergic NMDA Ca2 + flux in the hippocampus, critical for modulating the relative strength and efficacy between synaptic connections [67]. CB_1_R agonists also modulate synaptic plasticity by dysregulating the protein synthesis involved in the formation and degradation of synaptic connections [68]. Our findings extend this work to show that CB1R availability is not associated with glutamate levels in the anterior cingulate. Future work should investigate whether glutamatergic alterations in the striatum and hippocampus are also associated with CB_1_R alterations.

### Strengths and limitations

A strength of the study was that all participants had not been taking any compounds acting on the central nervous system, including pharmacological treatments or illicit substances. While we cannot exclude the possibility that prior use of antipsychotics may have influenced the results, patients had a minimum drug washout period of 6 months and no patients had previously taken depot medications.

Moreover, patients had negative urine drug screens prior to scanning using a test that was able to detect cannabis, cocaine, amphetamine, and opiate use, and we excluded subjects with a history of substance use or dependence. Prior substance use is, therefore, unlikely to be a significant confound. However, since occasional users may have THCCOOH concentrations below the limit of sensitivity (50 ng/mL) [69], infrequent cannabis use may have been undetected. While some volunteers had previously used cannabis, 1 month of abstinence has been shown to normalise CB_1_R levels [70].

While we cannot exclude the possibility that the correlation between CB_1_R availability and glutamate may have been underpowered, our sample size was comparable to previously published findings investigating the association between dopamine and glutamate in first episode psychosis [27]. While our sample size was powered to detect clinically significant associations (*r* > 0.4), it may have been underpowered to detect smaller associations. However, the clinical significance of smaller associations is unclear. While only males were included due to sex differences in CB_1_R [71], future studies are needed to determine if female patients show CB_1_R alterations and whether these may be linked to glutamatergic function.

Glutamate metabolite estimates acquired using proton magnetic resonance spectroscopy reflect the average signal of intra-cellular and extra-cellular glutamate levels within a particular brain region [72]. In view of the limited spatial resolution of proton magnetic resonance spectroscopy, these findings do not exclude the possibility that pre-synaptic synthesis of glutamate or release may be associated with cannabinoid 1 receptor levels. Previous work has shown that glutamate release can be modulated following the administration of a cannabinoid 1 receptor agonist in rodents [36, 37] and humans [38]. However, we may have failed to observe an association, because we measured the average of both intra-cellular and extra-cellular glutamate. In view of these limitations and that glutamatergic functioning is involved in the dynamic modulation of synaptic connections thought to underlie learning and memory [73, 74], our findings do not exclude the possibility that patients with first episode psychosis may show other alterations in glutamatergic function that are related to CB1 receptors. Although we used a well-validated 1H-MRS neuroimaging acquisition protocol, the voxel placement was not rotated to align the anterior cingulate, because GE scanners do not enable voxel rotation handles in their MRS prescription interface.

Future studies using 7-T scanners could be useful as higher field strengths improve the quantification of metabolites and improve signal-to-noise ratios. Previous literature has reported a 2.8 × increase in signal-to-noise ratios and a significant decrease in the variability of metabolite estimates acquired on a 7-Tesla scanner relative to metabolites acquired on a 3-Tesla scanner [75]. These methodological advances will improve the validity and reliability of metabolite estimates and enable us to estimate metabolites present at lower concentrations in the brain [75, 76].

### Implications

The radiotracer which we used, [^11^C]MePPEP, is not displaced by a synthetic analogue of an endogenous cannabinoid, methanandamide [77]. As such, the V_T_ of [^11^C]MePPEP is thought to primarily reflect receptor density, not receptor function. Our findings, therefore, indicate that the density of cannabinoid 1 receptor levels has not associated with glutamate levels in the anterior cingulate. However, these findings do not exclude the possibility that the function of the cannabinoid 1 receptor may be altered in patients, and that this may be associated with glutamatergic alterations. The function could be tested in the context of pharmacological challenges using cannabinoid 1 receptor agonists, shown to modulate glutamate levels in rodents [36, 37] and humans [38].

Since CB_1_R binding inhibits calcium entry into the pre-synaptic neuron via N-, P-, and Q-type calcium channels [32, 78], fewer CB_1_R may dysregulate calcium and potassium channels, leading to neurochemical alterations in psychosis [27, 79–82]. While we did not show that cannabinoid 1 receptor levels in the anterior cingulate were associated with glutamate levels in the same region, this finding does not exclude the possibility that cannabinoid 1 receptor levels may modulate of neurotransmitters implicated in psychosis including dopamine [83] and gamma-aminobutyric acid (GABA) [32]. Future studies are, therefore, needed to investigate if CB_1_R alterations precipitate other neurochemical alterations in psychosis.

## Conclusions

We did not find evidence of a relationship between cannabinoid 1 receptor levels in the anterior cingulate cortex and glutamate levels in the anterior cingulate cortex. However, exploratory analyses indicated that healthy volunteers showed a negative association between ACC glutamate and CB_1_R availability in the striatum and hippocampus; however, these associations were lost in patients. These findings highlight the need for future studies to investigate endocannabinoid signalling pathways, and to investigate whether they may be altered in schizophrenia.

## Electronic supplementary material

Below is the link to the electronic supplementary material.

Supplementary file1 (DOCX 238 kb)
