# Supplementary material for: Association between cannabinoid 1 receptor availability and glutamate levels in healthy controls and drug-free patients with first episode psychosis: a multi-modal PET and 1H-MRS study
Source: Eur Arch Psychiatry Clin Neurosci. 2020 Sep 28;271(4):677–87. doi: 10.1007/s00406-020-01191-2 (PMC8119269; doi:10.1007/s00406-020-01191-2)
Supplement: Supplementary file 1 — Supplementary file1 (DOCX 238 kb) [file 406_2020_1191_MOESM1_ESM.docx]

Supplementary materials


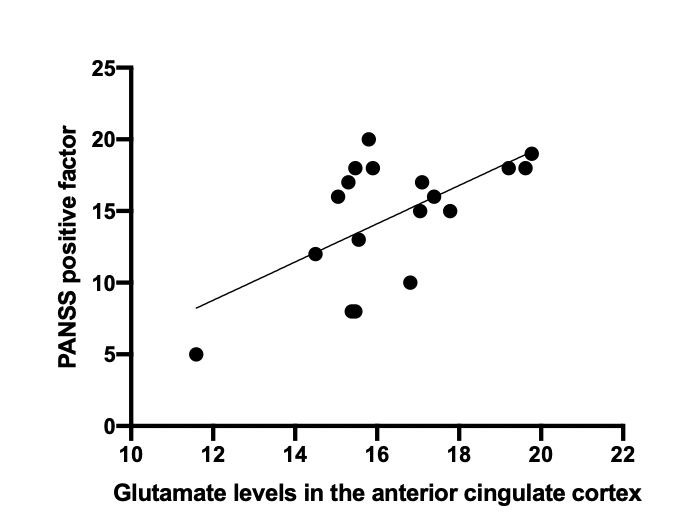


Supplementary figure 1. Association between positive factor, generated from a PANSS 5-factor model and glutamate levels in the anterior cingulate cortex (R=0.613, p=0.007).


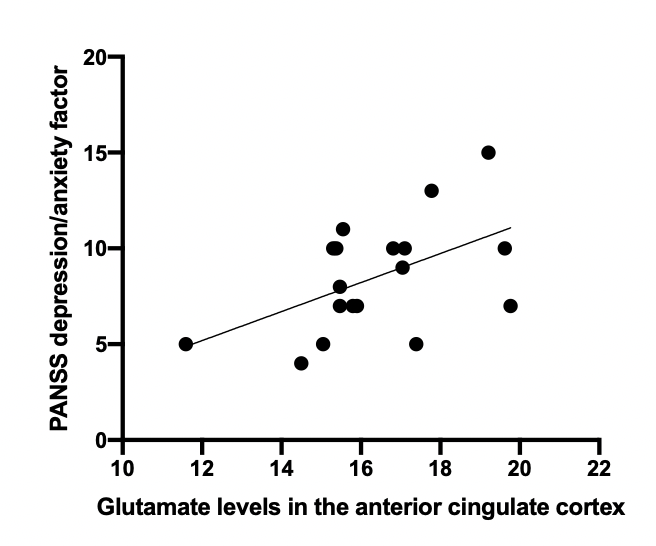


Supplementary figure 2. Association between depression/anxiety factor, generated from a PANSS 5-factor model and glutamate levels in the anterior cingulate cortex (R=0.514, p=0.029).
